# Autophagy and Macrophage Functions: Inflammatory Response and Phagocytosis

**DOI:** 10.3390/cells9010070

**Published:** 2019-12-27

**Authors:** Ming-Yue Wu, Jia-Hong Lu

**Affiliations:** State Key Laboratory of Quality Research in Chinese Medicine, Institute of Chinese Medical Sciences, University of Macau, Taipa, Macau, China; amywu1989@hotmail.com

**Keywords:** macrophage, autophagy, inflammatory response, phagocytosis

## Abstract

Autophagy is a conserved bulk degradation and recycling process that plays important roles in multiple biological functions, including inflammatory responses. As an important component of the innate immune system, macrophages are involved in defending cells from invading pathogens, clearing cellular debris, and regulating inflammatory responses. During the past two decades, accumulated evidence has revealed the intrinsic connection between autophagy and macrophage function. This review focuses on the role of autophagy, both as nonselective and selective forms, in the regulation of the inflammatory and phagocytotic functions of macrophages. Specifically, the roles of autophagy in pattern recognition, cytokine release, inflammasome activation, macrophage polarization, LC3-associated phagocytosis, and xenophagy are comprehensively reviewed. The roles of autophagy receptors in the macrophage function regulation are also summarized. Finally, the obstacles and remaining questions regarding the molecular regulation mechanisms, disease association, and therapeutic applications are discussed.

## 1. Introduction

Autophagy is a highly conserved mechanism by which the cytoplasmic cargo is delivered to the lysosomes for degradation. There are at least three forms of autophagy: chaperone-mediated autophagy (CMA), microautophagy, and macroautophagy. Macroautophagy is the major autophagic degradation form that maintains the cell homeostasis and organelle quality control in eukaryotic cells. Macroautophagy (hereafter referred to as autophagy) plays crucial roles in various cellular physiological processes, including cellular metabolism, residual cargo removal, renovation in cell differentiation and development. Emerging evidence has revealed the implications of autophagy in numerous diseases, including immunological diseases, cancer, neurodegenerative diseases, cardiovascular disorders and aging [1]. One feature of autophagy is the formation of a double membrane structure called autophagosome; this formation comprises four main steps: (1). autophagosome initiation, (2) autophagosome elongation, (3) autophagosome closure, and (4) autophagosome fusion with lysosome [2]. In the initiation stage, ULK1 complex (consisting of ULK1, FAK family interacting-protein of 200 kDa (FIP200), ATG13 and ATG101) is activated and localizes in the ER [3]. Subsequently, class III phosphoinositide 3-kinase (PI3K) complex (consisting of VPS34, VPS15, Beclin 1, ATG14L and NRBF2) is activated by ULK1 to generate PI3P which recruits its binding proteins, WD repeat domain phosphoinositide-interacting protein 2 (WIPI2), and zinc-finger FYVE domain-containing protein 1 (DFCP1) [4,5]. Then two ubiquitination-like systems are activated to elongate the autophagosome. In the first system, ATG5 is covalently conjugated to ATG12, and then interacts with ATG16L1. In the second system, ubiquitin E1-like enzyme ATG7, E2-like enzyme ATG3, and ATG5–ATG12 work together to facilitate the conjugation of phosphatidylethanolamine (PE) to the autophagosome-associated protein ATG8/LC3 [6]. ATG8/LC3 changed from LC3-Ⅰ form to LC3-II; form by conjugating with a phosphoethanolamine during autophagy. The membrane-bound LC3-II; induces the growth and closure of the autophagosome, which then fuses with lysosome via SNARE [7]. Recent studies have revealed that Vps21 regulates the ESCRT recruitment to the autophagosome, catalyzing its closure via working on a Rab5-controled Atg17-Snf7 interaction [8].

Macrophage is a type of white blood cell that professionally serves as phagocyte and antigen-presenting cell (APC). Originally, macrophages can be derived from progenitors in bone marrow and fetal precursors in the yolk sac. Macrophages originating from yolk sac-derived erythromyeloid progenitors (EMPs) are distributed to different tissues, such as skin (Langerhans cells), liver (Kupffer cells), brain (microglia), pancreas, lung and spleen (red pulp macrophage) and kidney [9,10]. During the homeostatic adaptations, tissue resident macrophages can also be refreshed via recruitment from monocytes in blood and bone marrow [11], or by local proliferation [12]. Macrophages are capable of clearing invading pathogen, triggering inflammatory signals and engulfing dead cells. Emerging evidence has revealed that, macrophages are necessary for different tissue growth and maintenance of metabolic homeostasis based on macrophagic phagocytosis and cytokine signaling regulation. Macrophages are involved in bone remodeling [13], brain development [14], controlling stem cell function [15,16], the regulation of angiogenesis [17,18], and remodeling of tissues [19,20]. In addition, macrophages regulate metabolic homeostasis of white and brown adipose tissues, liver and pancreas [21]. Macrophages plays a dual role during injury and pathogen invasion. In many diseases, such as cancer, inflammatory-related diseases and fibrosis, macrophages are regarded as contributing more to the disease progression when inflammatory macrophages cannot be suppressed [22,23,24,25]. However, macrophages also contribute to inflammation resolution via engulfing dead cells, and being triggered into M2-like macrophage state [26].

Accumulating evidence suggests that macrophage is one of the bridges connecting autophagy and immunity [27,28]. Autophagy regulates cellular development of monocytes, resulting in the disturbance of macrophage differentiation [29,30]. The activation of autophagy leads to the recycling of cellular components and ATP, which are exactly what macrophages requires in their energy architecture, especially during activation [31]. In addition, autophagy and phagocytosis in macrophage shared lots of genes during the process, such as *Beclin1, Vps34*, and *Atg5* [32]. In this review, we focus on the emerging evidence and the roles of the autophagic genes or their coding proteins in regulating macrophage function, highlighting how autophagy functions in inflammatory response and phagocytosis from different aspects in the angle of macrophages.

## 2. Autophagy and Macrophage Pattern Recognition Receptors (PRRs)

As components of the innate immune system, macrophages and other immune cells utilize pattern recognition receptors (PRRs) to identify invading pathogens by engaging pathogen-associated molecular patterns (PAMPs). PRRs can be subdivided into two kinds: cell surface receptors, and intracellular receptors. Toll-like receptors (TLRs), scavenger receptors, and lectins are cell surface receptors, while NOD-like receptors (NLRs) and RIG-1-like receptors (RLRs) are intracellular receptors. Recently, studies have revealed that autophagy can be regulated via activating PRRs.

## 3. Scavenger Receptors and C-Type Lectin Receptors

Scavenger receptors were initially thought to recognize modified low-density lipoprotein (LDL); however, currently they are known to bind to a variety of proteins or pathogens. Class A scavenger receptor (SR-A) and macrophage receptor with collagenous structure (MARCO) have been revealed to be engaged in autophagy regulation. SR-A activated by fucoidan inhibited autophagy and contributed to macrophage apoptosis [33]. MARCO, a receptor for recognition of un-opsonized or environmental particles, can be internalized to incorporated by extracellular materials into cells via the endocytosis–autophagy pathway [34]. C-type lectin was originally known to recognize carbohydrates in a Ca^2+^-dependent manner [35], and later it was shown to bind with many ligands including lipids, protein, or other molecules [36,37]. Dectin-1 is a C-type lectin and has been proven to regulate autophagy-dependent unconventional processes in macrophage, such as protein secretion [38] and LC3 dependent-phagocytosis [39].

### 3.1. Toll-Like Receptors (TLRs)

TLRs are type 1 integral transmembrane proteins that form a horseshoe shaped structure responsible for pathogen recognition. TLRs engagement leads to a ligand specific and TIR domain-dependent recruitment of adaptors proteins including myeloid differentiation factor 88 (MyD88) and TIR domain-containing adapter-inducing interferon-b (TRIF) [40]. Interestingly, many studies found that many TLRs can regulate autophagy. TLR1, 2, 3, 4, 5 and 7 can induce autophagosome formation during immune response [41,42,43]. Mechanistic study has revealed that MyD88 and TRIF interact with Beclin 1 as a TLR signaling complex component to facilitate autophagy induction by inhibiting the interaction between Beclin 1 and Bcl-2 [41]. Meanwhile, TRAF6, a key ubiquitin E3 ligase in the TLR pathway, binds with Beclin 1 and regulates its lysine (K) 63-linked ubiquitination for autophagy induction [44,45]. Therefore, Beclin 1 complex can be a mediator for the TLR-induced autophagy. TLR-induced autophagy can also be selective. After treatment of macrophages with *Escherichia coli* or lipopolysaccharide (LPS), TLR4 activated autophagy to selectively target aggresome-like induced structures (ALIS) with the assistance of p62 [46]. Collectively, TLRs are key players in the autophagosome formation during pathogen-invading, the real roles of TLR-induced autophagy in the regulation of macrophage function, however, have not been well characterized.

### 3.2. NOD-Like Receptors (NLRs)

NLRs are the key components of surveillance systems for the detection of intracellular pathogens. NOD1 and NOD2 are two well-described receptors in this family, which sense bacterial peptidoglycan and initiate proinflammatory responses [47,48]. NOD1 and NOD2 have been shown to induce autophagy initiation by interacting and recruiting ATG16L1 [49]. Coincidentally, polymorphisms in both NOD2 and ATG16L1 genes are associated with Crohn’s disease [50,51], which underscores the intrinsic connection between these two factors in biological function and human diseases. Moreover, further study has revealed that ATG16L1 suppresses NOD1- and NOD2-induced cytokine response via the induction RIP2 activation, but independent of autophagosome formation [52]. Therefore, autophagy induced by NOD2 is distinct from RIP2 or NF-κB pathways.

**Figure 1:**
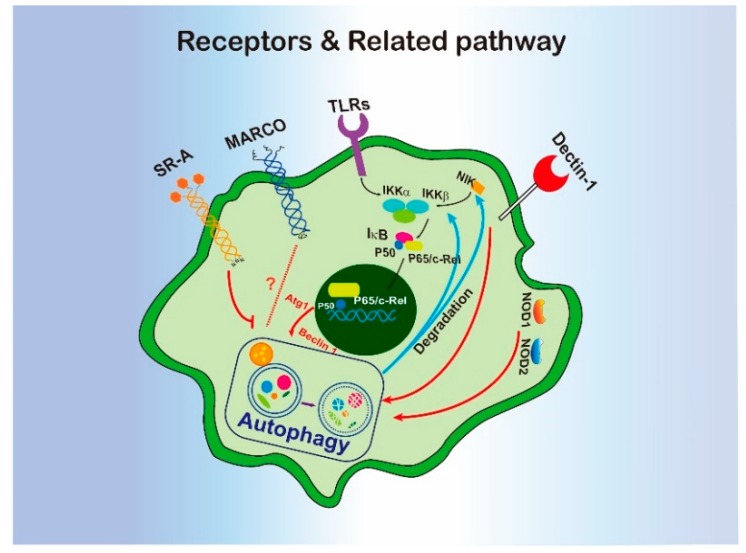
The interregulation of macrophagic receptors and autophagy.

The above evidence suggests that PRRs and autophagy closely interact with each other to regulate macrophage function (Figure 1). PRRs normally initiate signaling at the earliest stage of pathogen recognition. That is, they regulate autophagy mostly in the very beginning, for example, Beclin 1 directly binds to the MyD88 and TRIF [41]. The inhibition of autophagy suppresses PRRs-related biological functions, such as dectin-1-induced vesicle-mediated protein secretion [38], TLRs involved-bacterial clearance [42], and IFN-α secretion [53]. However, the mechanisms by which autophagy is regulated by PRRs and the consequences of autophagy induction for the anti-pathogenic function of macrophages still needs further exploration.

## 4. Autophagy and Inflammatory Pathways in Macrophages

### 4.1. Cytokines and Autophagy

Cytokines are secreted proteins produced mainly by macrophages and lymphocytes to mediate an effective immune response by influencing the inflammatory microenvironment [54,55]. Although, autophagy deficiency has been implicated in several inflammatory diseases, such as IBD [50,51], systemic lupus erythematosus (SLE) [56,57], and arthritis [58,59], the role of autophagy in inflammatory cytokine production is still not clear. The loss of *Atg7* results in the increased production of IL-1β and pyroptosisi after *P. aeruginosa* infection, however, IL-6 and TNF-α levels are not affected [60]. Administration of 3-methyladenine (3-MA) (an autophagy inhibitor) attenuated the sepsis symptoms as well as the IL-6 and TNF-α production in a lethal model of murine sepsis [61]. *Atg5^fl/fl^* lysM^−^Cre^+^ mice showed significantly increased IL-1α, IL-12, and CXCL1 in lung tissue after *M. tuberculosis infection*, but the universal pro-inflammatory cytokines, such as IFN-γ, TNF-α, and IL-6 were not affected [62]. Therefore, the role of autophagy in cytokine production is not a consequence of general inflammatory stimulation, but it involves more specific mechanisms.

### 4.2. NF-κB Pathway and Autophagy

NF-κB is a well-known transcription factor that regulates a large array of genes related to inflammatory response [63]. In macrophages, both pathogen-associated molecular patterns (PAMPs) and damaged-associated molecular patterns (DAMPs) can stimulate the NF-κB pathway to enhance cell survival, proliferation, inflammatory response, and angiogenesis by activating the production of multiple cytokines (IL-6, TNF-α, etc.), chemokines (MCP-1, IL-18, CXCL10, etc.), cell cycle regulators (Bcl-2L1, Cyclin, etc.) and adhesion molecules (ICAM-1, VCAM-1, etc.) [64].

Autophagy has been shown to regulate the degradation of NF-κB-inducing kinase (NIK) and of the essential activator of NF-κB, IκB kinase (IKK) under Hsp90 inhibition conditions [65,66]. In fact, emerging evidence has confirmed that alteration of NF-κB pathway regulates autophagy level. In 2007, Mojavaheri-Mergny., et al. found that the NF-κB pathway activation represses TNF-α-induced autophagy in different cancer cell lines [67]. Later, a NF-κB binding site in the promoter of the *BECN1* was identified, and NF-κB family member p65/RelA was shown to upregulate *BECN1* mRNA expression to activate autophagy [68]. Recently, the NF-κB factor Relish has been found to regulate autophagy by modulating ATG1 expression, thereby facilitating salivary gland degradation in *Drosophila* [69]. Meanwhile, the NF-κB pathway requires the activation of autophagy to mediate the degradation of the kinases involved in NF-κB activation to limit the inflammation cascades.

### 4.3. RIG-1 or STING Sensing Pathways and Autophagy

RIG-1-MAVS and cGAS-STING are pathways to sense cytosolic RNAs or DNAs in pathogenic viral genomes to induce innate immune responses. RIG-1 like receptors (RLRs) can detect single-stranded and double-stranded RNAs that are generated after viral infection. This receptor family contains three elements: RIG-1, melanoma differentiation associated gene 5 (MDA5), and laboratory of genetics and physiology 2 (LGP2) [70]. In uninfected cells, RIG-1 binds to the adaptor protein MAVS on the surface of mitochondria. Once RIG-1 is activated, the MAVS forms aggregates and converts its structure into functional multimeric filaments, stimulating recruitment of numerous adaptor proteins and kinases [71]. Eventually, RIG-1 signaling is cleaved into two molecular cascades: TANK binding kinase-1 (TBK-1) and IκB kinase epsilon regulating the production of type 1 and type iii IFNs; the other cascade is engagement of the IKKα/β/γ complex and activation of NF-κB [72].

Roles of autophagy in the RIG-1-MAVS sensing pathway has been reported in recent years, but it’s difficult to figure out the process based on the different studies. In the early study, ATG5-ATG12 conjugate was shown to regulate the RLR signaling. Replication of VSV virus was inhibited in *Atg5*-deficienct MEFs, accompanied by increased IRF-3 activation and IFN-β transcription, accumulation of dysfunctional mitochondria, increased ROS, and RLR signaling [73,74]. A later study also revealed that ATG5, ATG12, and ATG7 are all required for the negative regulation of RLR signaling, in which ATG5–ATG12 conjugate interacts with RIG1 and MAVS to inhibit RLR-dependent antiviral signaling [75]. However, in a recent study, activation of the RIG-1 RNA sensing pathway was appeared to trigger autophagy via the MAVS-TRAF6-Beclin-1 signaling Axis [76]. Collectively, autophagy regulation in RIG-1-MAVS pathway is not consistent in different conditions, and these inconsistencies may come from the distinct viruses studied or the different regulatory functions of autophagy-related proteins.

CGAS-STING is a pathway to distinguish non-self DNA from pathogens and induce innate immunity responses. cGAS is a central regulator of cytosolic DNA sensing [77]. When cGAS is activated, cGAS stimulates generation of the second messenger, cGAMP. Subsequently, cGAMP activates 2′-5′ phosphodiester linkages to bind with the downstream signaling molecule STING on the ER membrane. Finally, STING binds to TBK1 to drive IRF3 activation, thereby inducing IFNβ upregulation [78,79].

A study in 2012 revealed a link between DNA sensing and autophagy in macrophages infected by *Mycobacterium tuberculosis* (*Mtb*). The recognition of bacterial DNA via the STING pathway induced autophagy [80]. Further, cGAMP also induced the activation of autophagy kinase, ULK1, to phosphorylate STING and prevent the downstream signaling [81]. Interestingly, STING pathway can also be regulated by autophagy via TBK1-mediated p62 phosphorylation to drive STING ubiquitination and autophagic degradation [82].

**Figure 2:**
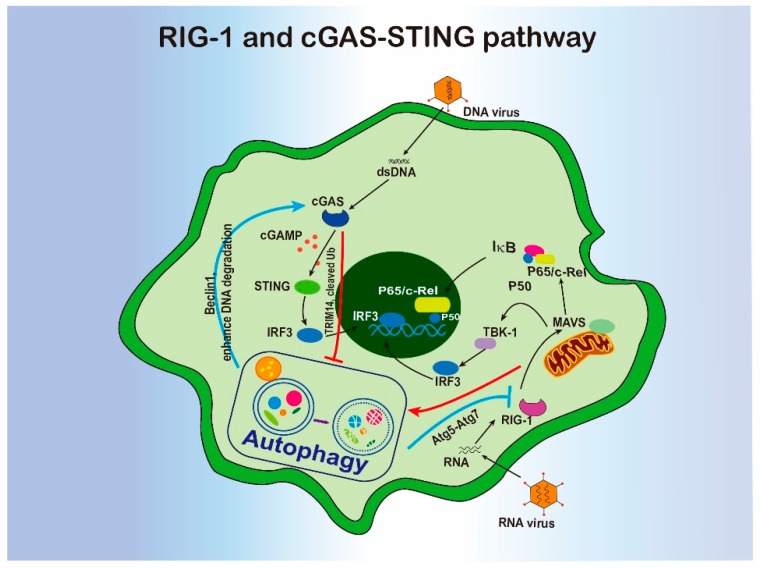
The interregulation of RIG-1 or cGAS-STING and autophagy.

Later study found that cGAS DNA sensor was able to directly interact with Beclin-1 to halt cGAMP production and enhance the degradation of cytosolic microbial DNAs via autophagy [83]. In addition, TRIM14, a E3 ligase, recruited USP14 to stabilize cGAS by cleaving the lysine 48-linked ubiquitin and inhibiting the p62-mediated autophagic degradation of cGAS to increase type I interferon signaling [84]. Most recently, STING was found to activate autophagy to eliminate DNA and viruses in the cytosol [85]. STING-containing ER-golgi intermediate compartment (ERGIC) is a membrane source for autophagic LC3 lipidation. The activated LC3 lipidation is dependent on WIPI2 and ATG5 but independent of ULK and the VPS34-beclin 1 complex. In summary, autophagy has diverse roles in the entire SITNG pathway and restricted autophagy-related proteins are responsed in the pathway (Figure 2).

### 4.4. Inflammasome Pathway and Autophagy

Besides the NF-κB pathway, inflammasome formation is another mechanism to regulate innate immunity in macrophages. Activation of inflammasome leads to maturation of the proinflammatory cytokines IL-1β and IL-18 by activation of the proteolytic enzyme caspase-1 [86]. Similar to NF-κB, inflammasomes can be triggered by a wide range of stimuli, including PAMPs and DAMPs [86]. Inflammasomes localize at cytoplasma and are assembled by multiple proteins [86].

In terms of components, inflammasomes contain a cytosolic sensor protein (which can be either a nucleotide-binding oligomerization domain and leucine rich repeat-containing (NLRs) protein, or an AIM2-like receptor (ALR) protein), an adaptor protein (apoptosis-associated speck-like (ASC) protein that containing a caspase-recruitment domain (CARD)), and an effector pro-caspase-1 [87]. ASC is a protein that contains both N-terminal Pyrin domain (PYD) and a CARD. Until now, several inflammasomes have been identified, including NLRP1, NLRP3, AIM2 and NLRC4. NLRP3 inflammasome is comprised of NRLP3, ASC, and pro-caspase-1. In NLRP1 inflammasome, NLRP1 protein already contains both PYD and CARD which can interact with pro-caspase-1 directly without ASC. AIM2 inflammasome contains AIM2 protein, ASC and pro-caspase-1. NLRC4 inflammasome contains a sensor protein with CARD domain and pro-caspase-1 without ASC.

In 2008, Tatsuya Saitoh et al., reported the relationships between autophagy and inflammasome. They found that *Atg16L1* deficient macrophage enhances IL-1β and IL-18 production but not LPS-induced IL-6 or TNF-α production [88], leading to subsequent studies that uncovered the underlying mechanisms. Mitochondrial-derived DAMPs are stimuli for inflammasome activations. Autophagy has been shown to remove these DAMPs, thereby preventing inflammasome activation. Disruption of autophagy exaggerated NLRP3 inflammasome activation in response to stimuli in macrophage, accompanied by the increase in the ROS-producing mitochondria [89,90]. In addition, the released mitochondrial DNA (mtDNA) is also responsible for stimulating NLRP3 inflammasome [71]. Autophagy is responsible for the supervision and efficient clearing of dysfunctional mitochondria, preventing mitochondrial-derived DAMP-induced inflammasome activation.

**Figure 3:**
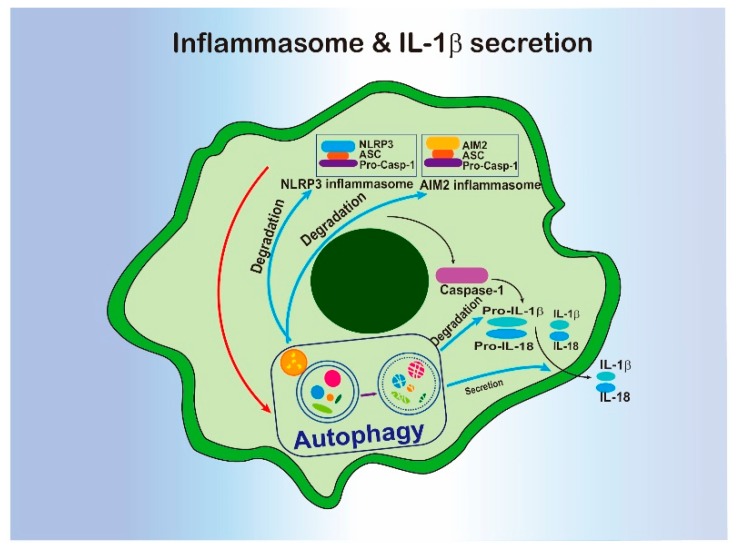
The interregulation of inflammasome and autophagy.

P62 is a key receptor in the selective autophagical clearance of inflammasome and dysfunctional mitochondria during inflammasome pathway activation. P62 is recruited to K63 (Lys63)-linked polyubiquitination of ASC, thereby inducing ASC-targeted autophagosome formation and degradation [91]. AIM2 is associated with tripartite motif11, an E3 ubiquitin ligase, to facilitate p62 recruitment for autophagic degradation [92]. As inflammasomes are platforms composed of multiple proteins, autophagy plays a vital role in the degradation of these large protein complexes. Collectively, autophagy regulates the inflammasome pathway primarily via mediating the degradation of either the activator of or the components of inflammasome. Interestingly, Zhenyu Zhong et. al, reported that NF-κB exerted anti-inflammatory activity by inducing p62 expression to accelerate mitophagy and reduce inflammasome activation [93]. These findings revealed a complicated regulation network between autophagy and inflammasome pathways (Figure 3).

### 4.5. Macrophage Polarization

Macrophages are heterogeneous and can be polarized into divergent phenotypes in different tissue microenvironments. Typically, macrophages can be divided into M1 phenotype (classically activated) and M2 phenotype (alternatively activated). Interferon-γ and lipopolysaccharide (LPS) can induce the M1 phenotype with increased pro-inflammatory cytokine production and cellular immunity, while IL-4 or IL-13 can activate the M2 phenotype to promote tissue repair and humoral immunity [94,95].

The role of autophagy in regulating macrophage polarization has been reported to be mediated by different mechanisms including NF-κB degradation and the mTOR pathway [96,97,98]. Although NF-κB can be activated after M1 polarization, in fact, activation of NF-κB is able to drive macrophages to either M1 or M2 polarization especially in the tumor microenvironment [99,100,101]. In 2013, Chih-Peng Chang et al. found that TLR2 signal induces NF-κB p65 cytosolic ubiquitination which results in its degradation by p62-mediated autophagy. Meanwhile, NF-κB activity rescued by inhibiting autophagy drove macrophages to the M2 phenotype [96,97]. Further, mTOR is the master controller of autophagy and is involved in regulation of macrophage polarization. Previous studies found that mTOR pathway activation induces macrophage polarization. Rapamycin, a well-known autophagy inducer which acts via inhibiting the mTOR pathway has been shown to stimulate M1 phenotype of macrophages [98]. Blockage of the mTOR pathway by silencing TSC2 (tuberous sclerosis 2) produced the opposite effect [98]. Besides NF-κB and mTOR pathways, there is accumulating evidence of other potential relationships between autophagy and macrophage polarization. For example, CCL2 and IL-6 are potent factors to induce autophagy in macrophages, and they can trigger the M2 phenotype [102]. Sorafenib, a multi-kinase inhibitor, has been shown to induce autophagy and suppress macrophage activation via inhibiting the expression of macrophage surface antigens [103].

Inducible nitrogen oxidase (iNOS) and Arginase 1 are two enzymes that are the markers of macrophage M1/M2 polarization. M1 macrophages express iNOS and metabolize arginine to nitric oxide (NO) and citrulline, while M2 macrophages hydrolyze arginine to ornithine and urea. Some studies have uncovered a potential relationship between autophagy and the expression of iNOS and arginase. Further, miR-326 overexpression inhibits iNOS expression and also promotes autophagy [104]. Antimicrobial autophagy has been suppressed by glucocorticoids, accompanied by the enhancement of iNOS expression and NO production [105]. Activation of autophagy in LPS-stimulated microglia suppresses the iNOS expression [106]. Interestingly, recombinant human arginase induces autophagy [107].

**Figure 4:**
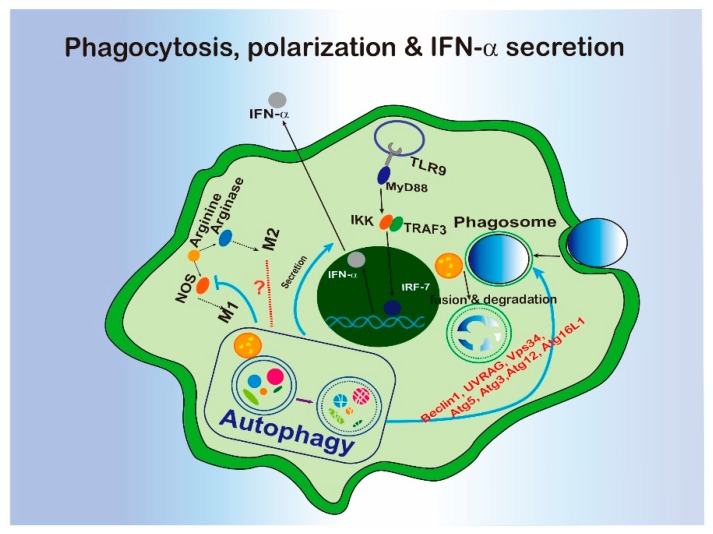
The interregulation of phagocytosis, macrophage polarization, or IFN-α secretion and autophagy.

Although autophagy has been shown to be involved in the regulation of macrophage polarization, most evidence is not clear as to the mechanism by which autophagy regulates macrophage polarizations (Figure 4). Whether an autophagy gene directly modulates the signaling pathways for macrophage polarization, or whether autophagy regulates the degradation of key proteins involved in macrophage polarization regulation, will need to be confirmed.

### 4.6. Cytokine Secretory Pathway

Cytokines and chemokines act as messengers to orchestrate immunity when secreted out from producing cells, such as macrophages. There are two types of cytokine release by secretion: conventional secretion, and unconventional secretion. Conventionally, cytokines with an N-terminal signal peptide enter the endoplasmic reticulum (ER) and follow a secretory pathway via the Golgi apparatus for delivery to the extracellular space [108]. However, some cytokines lacking signal peptides cannot enter the ER, and they rely on unconventional secretion pathways, including autophagy [109], for their release.

IL-1β, IL-18 and IL-15 are cytokines that lack signal peptide entering the ER [110]. Among them, IL-1β has been extensively studied in recent years. Accumulating results show that IL-1β trafficking is closely related to autophagy. For example, IL-1β can be sequestered into autophagosomes for degradation, and induction of autophagy by rapamycin blocks mature cytokine secretion and activates the pro-IL-1β degradation [111]. Following this discovery, TRIM16 was identified as the secretory autophagy receptor for IL-1β that interacts with R-SNARE SEC22B to delivery IL-1β to the LC3-positive sequestration membranes [112]. However, the autophagosome has also been shown as a mean to promote IL-1β secretion in neutrophils [113]. In addition to IL-1β, but IFN-α production has also been reported to rely on the LC3-associated phagocytosis in TLR9-related trafficking [53] (Figure 4). Taken together, the roles of autophagy in regulating secretory pathways for cytokines are not fully understood, and it seemed to be different in distinct cell types. Autophagy mediates IL-1β generation by facilitating pro-IL-1β degradation. It is still challenging to figure out the key regulatory points that connect autophagy and cytokine secretion.

## 5. Autophagy and Macrophage Phagocytic Function

### 5.1. LC3-Associated Phagocytosis

Phagocytosis is defined as a pathway for the recognition and internalization of particles (>0.5 μm) by the professional phagocytes, such as macrophages or non-professional phagocytes, such as epithelium cells [114] to maintain the cell homeostasis when threatened by invaders’ attacking [114]. In 2007, the Douglas R. Green Lab firstly reported that Beclin 1, LC3, Atg5 and Atg7 can be recruited to the phagosome in macrophage during TLR pathway activation [115]. This process has been identified as unconventional LC3-associated phagocytosis (LAP). The characteristics of the LAP molecular requirements have been revealed. Rubicon is required for the activity of a Class III PI3K complex containing Beclin-1, UVRAG, and Vps34 during LAP to generate PI (3) P without ATG14 and Ambral, two conventional autophagy-related proteins. Further, ATG5, ATG3, ATG12, and ATG16L are all necessary for the conjugation of LC3 to the LAPosome [32] (Figure 4).

Based on the biological functions of phagocytosis, LAP displayed various roles in immune response (Figure 4). For example, LAP activation has been connected to innate immune recognition, or the killing of pathogens. LAP promotes antigen presentation by MHC class II molecules to T cells [116] and helps to clear pathogens via engulfment and phagosome acidification [32,115]. In terms of the inflammatory response, LAP has been implicated in DNA-induced TLR9 pathway activation. LC3 and kinase IKKα have been found to form a complex to be recruited to the endosome containing TLR9, and these complex are further associated with TRAF3 and IRF7 with the help of ATG5 to promote type 1 interferon production [117]. In the tumor microenvironment, defects of LAP induced control of tumor growth by tumor-associated macrophage (TAM) through triggering pro-inflammatory gene expression and triggering a STING-mediated type I interferon response [118]. Since a series of autophagy-related proteins are involved in LAP, LAP could be a major link between autophagy and innate immunity.

The role of LAP in dead cell clearance was reported in 2011 [119]. The study showed that LAP can be evoked by incubating macrophages with apoptotic, necrotic and RIPK3-dependent necrotic cells. Dead cells were efficiently degraded through LAP. As the defective clearance of dying cells is associated with systemic lupus erythematosus (SLE), this study found mice with LAP pathway defects displayed increased SLE-like symptoms after the repeated injection of apoptotic cells [120].

### 5.2. Selective Autophagy in Macrophage

Autophagy was initially regarded as a non-selective process, while recent studies revealed that autophagy can also be selective. Selective autophagy is responsible for selectively removing of specific cellular cargos, by a recognition mechanism involving autophagy receptors or adaptors [121]. According to different cargos, selective autophagy has been classified into different types, such as aggrephagy (cargos: protein aggregtes), mitophagy (cargos: mitochondria), xenophagy (cargos: pathogens); ER-phagy (cargos: endoplasmic reticulum), pexophagy (cargos: peroxisomes), and so on.

### 5.3. Xenophagy

Xenophagy is a form of selective autophagy which specifically targets invading pathogens. Xenophagy in macrophage has been well characterized during *Mycobacterium tuberculosis* infection. Specifically, the internalized phagosomal *M. tuberculosis* can be released into cytosol by damaging the phagosomal membrane [80]. Escaped bacterial DNA was recognized by cytosolic sensor cGAS, which induced xenophagy via activating ubiquitination by ubiquitin ligases Parkin and Smurf1 and recruiting autophagy receptors or adaptors, such as p62 and NDP52 [122,123,124]. Not only the bacterial but also damaged phagosomes containing bacteria can be targeted by the host glycan on the phagosomal lumen [125,126]. Ultimately, bacteria were sent to the lysosome via the autophagosome for degradation. In this process, autophagy also facilitated the host to kill bacteria via generating and delivering antimicrobial peptides to the compartment [127,128]. In addition, NOD1 and NOD2 sensed invasive bacterial and induced xenophagy by recruiting Atg16L1 to the site of bacterial entry [49]. The adaptors or receptors for xenophagy are not limited to p62 and NDP52, neighbors of the *BRCA1* gene 1 (NBR1) and optineurin can also serve the xenophagy process [129].

**Figure 5:**
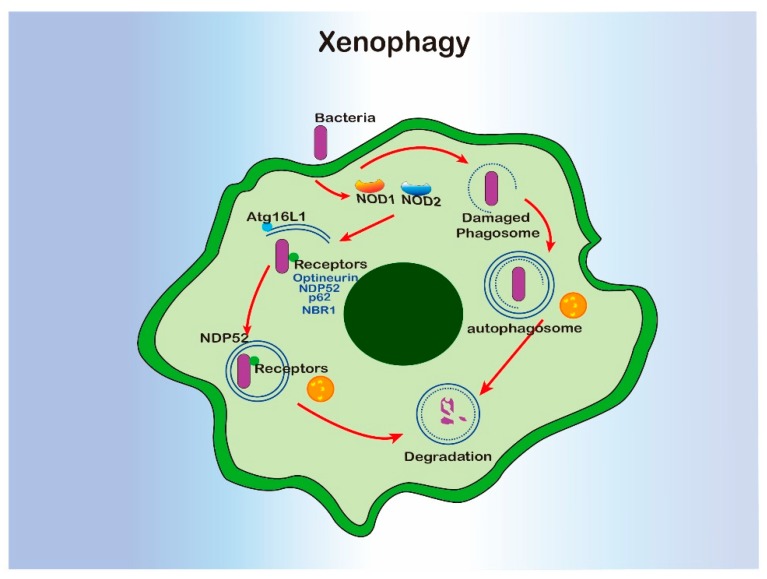
The interregulation of xenophagy and macrophage.

Even though xenophagy requires most of the molecular machinery involved in classical autophagy, increased susceptibility to *M. tuberculosis* infection was only observed in the mice with *Atg5* deficiency in monocyte-derived cells and neutrophils, not those lacking *Beclin-1* or *Atg14* [62,80,130]. In some cases, pathogens are able to develop various strategies to destroy autophagy for survival. For example *S. flexneri* can escape from xenophagy by secreting IcsB, which can inhibit bacterial recruitment to the phagophore via binding competitively to the surface protein VirG [131]. *L. monocytogenes* inhibits xenophagy via recruiting the Arp2/3 complex and Ena/VASP to the bacterial surface, where it masks the cell surface by binding to the cytoplasmic major vault protein (MVP) or blocking the lipidation of LC3 [132,133,134]. *Salmonnella typhimurium* secrets more than 30 effector proteins, resulting in the mTOR activation [135], deubiquitination of aggregates [136], and disrupting Rab1-A signal [137], and ultimately, autophagy suppression. In their most recent study, Shao Feng’s lab found that a novel protein, SopF, generated by Salmonella inhibit xenophagy by targeting the Gln124 of ATP6V0C in the V-ATPase, resulting in disruption of V-ATPase-ATG16L1 axis in xenophagy initiation [138]. So, xenophagy partially facilitates macrophage phagocytosis to clear invasive pathogens, especially when the phagosome has been damaged (Figure 5). However, xenophagy can also be blocked by flexible escape strategies developed by invaders.

### 5.4. Autophagy Receptors in Macrophage

Autophagy receptors are adaptor proteins which recognize cargos and bind to LC3/GABARAP on autophagosome [121]. The selectivity of cargos is mainly achieved by the specificity of autophagy receptors in recognizing distinct cargos. As mentioned above, p62 is a key autophagy receptor involved in the inflammasome pathway regulation. In addition, p62 and other receptors or adaptors also regulate macrophage functions via modulating selective autophagy process.

Selective receptors are involved in the innate inflammation pathway. According to the multiplex proteomic profiling, p62 and Tax1BP1 were identified to be the autophagy receptors that mediated the turnover of innate adaptor TRIF and its downstream signaling in *Atg16l1* deficient macrophages [139]. Knockdown of *TAx1BP1* increased cytokines release, such as IFN-β and IL-1β [139]. TRIM20 and TRIM21 are subsets of tripartite motif (TRIM) proteins, and they also acted as autophagic receptors to recognize inflammasome components or dimeric form of IRF3, delivering them for autophagic degradation [140]. As above mentioned, cGAS could also be degraded via autophagy after being recognized by p62. Conversely, p62-mediated autophagic degradation of cGAS enhanced the activation of type interferon I signaling [84]. Under LPS-induced inflammation, p62 interacts with iNOS and facilitates autophagic degradation of iNOS [141]. In cases of inflammasome pathway activation, p62 usually acted as an important receptor for inflammasome or related proteins degradation and so that to regulate inflammation response [91,92]. Inflammasome pathway can be easily activated by released mitochondrial DNA, and p62 has been reported to be involved in the inflammasome modulation as a receptor of mitophagy. P62-dependent mitophagy dysfunction caused inflammasome-induced IL-1β-dependent inflammation [93].

Several selective receptors are involved in xenophagy to mediate innate immunity response in macrophage. P62 and NDP52 are two classic receptors implicated in xenophagy. P62 assembles on the microbes as receptors once the cells were affected by pathogens, such as *Salmonella* [142] and mycobacteria [143]. NDP52 can be recruited as xenophagy receptors for *Salmonella* [144]. NDP52 has been proved to recruit ULK complex to the cytosol-invading bacteria and initiate autophagy [145]. Optineurin (OPTN) is another autophagy receptor that is implicated in xenophagy and controlling of TNF, NF-κB, and IFN signaling in macrophage [146,147,148]. TBK1 regulates OPTN phosphorylation to recognize cytosolic *Salmonella enterica* and trigger xenophagy [149,150]. During innate immunity response, OPTN served as a negatively regulator of NF-κB by promoting the xenophagy [151,152]. Another autophagy receptor, NBR1, was found to bind with viral capsid protein and particles of cauliflower mosaic virus (CaMV) for autophagic degradation [153]. TRIM5, a well-known retroviral restriction factor, was proposed to be a selective autophagy receptor targeting HIV-1 capsids for autophagic degradation [154]. Recently, V-ATPase was identified as the sensor of invading pathogen to recruit ATG16L to initiate xenophagy [138].

### 5.5. Autophagy and Macrophagic Metabolism

Emerging studies have revealed the crucial role of metabolic reprogramming in macrophage activation, which is known as immunometabolism [155]. For example, in amino acid metabolism, arginine is converted to NO by iNOS in M1 macrophage, but metabolized by arginase-1 in M2 macrophage [156,157]. M1 macrophage shows enhanced glycolytic metabolism and impaired mitochondrial oxidative phosphorylation (OXPHOS) [158,159]. In addition, the ATP produced in M1 cells via glycolytic metabolism feeds the pentose phosphate pathway (PPP) [160]. Fatty acid synthesis (FAS) organizes the plasma membranes under different inflammatory responses to regulate the inflammatory signaling, adhesion, and migration of macrophages [161]. Fatty acid oxidation (FAO) is needed for NLRP3 inflammasome activation [161]. These studies highlight the view that macrophagic metabolism status is tightly associated with macrophage functions.

Autophagy is an important part of cellular metabolism system that can be activated to supply materials and energy during nutrient deficiency. Therefore, the relationship between autophagy, metabolism, and macrophage function is a hotspot being noticed and investigated. In 2018, the “Autophagy, Inflammation, and Metabolism” (AIM) Center at the University of New Mexico has been established to specifically study this topic (ref: Autophagy, Inflammation, and Metabolism (AIM) Center of Biomedical Research Excellence). Further, mTOR functions as a key homeostatic regulator in nutrient signals and metabolic processes for cell growth [162], and its inhibition induce autophagy. As mentioned in the previous part, the mTOR pathway also regulates macrophage inflammatory pathway and polarization, which could be a possible link between cellular metabolism and macrophage function. AMP-activated protein kinase (AMPK) is a main sensor of cellular energy status and maintain metabolic balance [163]. AMPK can control cell growth by inhibiting mTOR pathway via phosphorylation of TSC2 or Raptor [164,165]. Therefore, AMPK can be involved into autophagy indirectly through acting on mTOR pathway. In addition, AMPK can also phosphorylate ULK1 complex directly to activate autophagy [166]. According to previous studies, AMPK is regarded as a suppressor of inflammation in immune cells including macrophage [167], indicating a potential connection among autophagy, cellular metabolism and macrophage inflammation. Besides, growing evidence has revealed that autophagy can regulate immune cell differentiation by alteration of metabolic states in immune cells [168]. Collectively, upon mTOR activation, immune cells show decreased autophagy, but increased cellular glycolytic and pro-inflammatory response. However, AMPK induction induce autophagy, increase cellular OXPHOS and anti-inflammatory response [168]. Moreover, increased lipophagy (autophagic target on lipid specifically) has been proved to accelerate macrophage cholesterol efflux so that to reduce macrophage foam cells in atherosclerosis [169]. Although lots of evidence is being uncovered, the regulation network between autophagy, metabolism, and macrophage function has not been well understood. The key metabolic products that link macrophage function and autophagy would be an interesting topic to explore

## 6. Conclusions and Discussion

As an automatous system of cellular defense, autophagy is naturally involved in innate immunity. An accumulation of evidence has revealed the intrinsic interaction between autophagy and macrophage function: Autophagy is involved in almost all macrophage functions, from pathogen recognition and phagocytosis to cytokine release and inflammatory responses (Table 1). However, there are still many unanswered questions. Cellular metabolism status changes dramatically during macrophage polarization. Autophagy is a cellular bulk metabolism mechanism. It would be interesting to examine whether metabolism change is a general link between autophagy and macrophage polarization. Autophagy is essential for LAP to degrade many cargos including bacterial, dead cells soluble ligands and protein aggregates. However, it is still unclear how LC3 is recruited to the phagosomes and which receptors are responsible for recognizing the different cargos. Selective autophagy, especially xenophagy, facilitates macrophages to eliminate the cytosolic invaders after pathogenic DNA exposure or phagocytic membrane damaged by pathogens [80,125,126]. Autophagy receptors have been reported to mediate inflammatory signaling and xenophagy by specifically recognizing the cargos for autophagic degradation. However, what are the signals on the cargos to recruit autophagy receptors? How are these receptors regulated to recognize different cargos? Is there any unknown autophagy receptor involved in inflammation regulation? Is autophagy defect associated with multiple inflammatory diseases, such as IBD, SLE, and arthritis. However, the deficiency in different autophagy genes sometimes generates quite different phenotypes in inflammatory response, raising the question of whether some autophagy proteins function via their autophagy-independent activity, or whether different stages of autophagy (or different groups of autophagy proteins) differentially regulate inflammatory responses. A more important question is whether we can use knowledge of how to regulate autophagy function in macrophages for diagnostic or therapeutic purposes, especially with regard to inflammatory conditions. With the continued, dedicated exploration of the mechanistic regulation between autophagy and macrophage function, hopefully, answers to the above-mentioned questions can be found.

## Figures and Tables

**Table 1:** The autophagy-related proteins involved in macrophage functions.

Category	Functional Proteins in Macrophages	Autophagy-Related Proteins	Mechanism or Relationships	Reference
Macrophage Receptors	SR-A	LC3	SR-A agonist inhibits autophagy by inhibiting LC3	[33]
	MARCO	LC3-ll/LC3-l	MARCO is internalized as phagocytosis, accompanied by increased LC3-ll/LC3-l	[34]
	Dectin-1	Atg3, Atg7, DAP1, GABRAPL2, Optineurin, RGS9, P62	Autophagy process is activated and required in Dectin-1 induced unconventional protein secretion pathway.	[38]
		LC3, Atg5	Dectin-1 directs LC3 and ATG5 recruitment around phagosomes to facilitate MHC class ll into phagosome and help antigens presentation to T cells.	[39]
	TLRs	LC3, Beclin 1	Interactions of Beclin 1 and Myd88 or Trif are increased after TLRs activation, so as to reduce the binding of Beclin 1 and Bcl-2, inducing autophagy. TRAF6 ubiquitinates Lysine (K)-linked ubiquitination of Beclin 1 and stimulates autophagy	[41,42,43,44,45]
		P62	LPS-induced TLR activation generates aggresome-like induced structures (ALIS), resulting in the recognition of p62 to induce autophagy	[46]
	NOD1, NOD2	Atg16L1	Interaction of NOD1 or NOD2 with Atg16L1 initiates autophagy	[49]
NF-κB pathway	NF-κB	Beclin 1 Atg1	NF-κB binding site is in the promoter of BECN1; p65/RelA upregulates BECN1 mRNA to activate autophagy. NF-κB factor Relish regulates autophagy by modulating Atg1	[68,69]
	IκB kinase (IKK)	Atg5	Autophagy mediates IKK protein degradation after inhibiting Hsp90 by geldanamycin (GA).Inhibition of autophagy by knockout of Atg5 rescues IKK from GA-induced IKK degradation.	[62]
	NIK	Atg5	Autophagy mediates NIK protein degradation after inhibiting Hsp90 by geldanamycin (GA). Blockage of autophagy by knockout of Atg5 inhibits GA-triggered NIK degradation.	[63]
Inflammasome	NLRP3	ULK1, P62	Mitophagy is induced by knockout of Ripk2 by increasing the phosphorylating ULK1, which activates NLRP3 inflammasome. Palmitate induces the activation of NLRP3 inflammasome with increased ULK1 dependent mitophagy. NLRP3 inflammasome activation triggers autophagosome formation, and it assembles inflammasomes undergo ubiquitination and recruitment of p62 for delivery to autophagosomes. NLRP3 inflammasome is inhibited by clearance of stimuli via increased NF-κB-induced P62-dependent mitophagy.	[71,89,90,93]
	ASC	P62	P62 is recruited to K63-linked ubiquitination of ASC for inducing ASC-targeted autophagosome formation and degradation.	[91]
	AIM2	P62	AIM2 interacts with E3 ubiquitin ligase to facilitate P62 recruitment of autophagic degradation.	[92]
	IL-1β	LC3	Pro-IL-1β is sequestered into autophagosome for autophagical degradation, so that to inhibit mature IL-1β generation and secretion. IL-1β works with TRIM16 and R-SNARE SEC22B and is delivered into LC3-positive autophagic membranes for secretion. IL-1β secretion is increased after autophagy activation with the augmented colocalization of IL-1β and LC3, but it will be decreased when autophagy is inhibited.	[111,112,113]
Polarization	iNOS	LC3, Atg5	MiR-326 upregulation promotes autophagy and downregulates iNOS expressioin in mice brain. Glucocorticoids inhibit iNOS expression and induces autophagy. Activation of autophagy suppresses iNOS expression in microglia; Depletion of Atg5 increases iNOS expression.	[104,105,106]
	Arginase	LC3, P62	Hepatoma tumor cell condition medium induces macrophage M2-like phenotype (increased Arginase 1 expression), accompanied by increased selective autophagy. Recombinant human arginase (rhArg) induces autophagy in lymphoma cells.	[97,107]
	CD206	LC3	CCL2 or IL-6 induces autophagy, companying with the macrophage polarization toward CD206^+^ M2-type activation.	[102]
TLR 9 pathway	IFN-α	LC3, Atg7	IFN-α secretion is required for LC3-associated phagocytosis (LAP).	[53]
	IKKα, IRF7 and TRAF3	LC3, Atg5	LC3 interacts with IKKα after TLR9 activation, and it is further associated with IRF7 and TRAF3 to facilitate type 1 IFN production.	[98]
LC3-associated phagocytosis (LAP)	PI(3)P	Beclin1, UVRAG, Vps34, Atg5, Atg3, Atg12, and Atg16L1	Autophagical genes, such as *Beclin1, UVRAG, Vps34, Atg5, Atg3, Atg12* and *Atg16L1* are required for LAP to generate PI (3) P and facilitate maturation.	[32]
Xenophagy	cGAS, Nod1 and Nod2,	Atg16L1, LC3, NDP52, p62, optineurin, NBR1, V-ATPase	Escaped bacterial DNA was recognized by cytosolic sensor cGAS, subsequently, inducing xenophagy via recruiting p62 and NDP52. NOD1 and NOD2 sense invasive bacterial and induced xenophagy by recruiting Atg16L1 at the site of bacterial entry. Neighbor of *BRCA1* gene 1 (*NBR1*) and optineurin also serve the xenophagy process. V-ATPase senses the membrane damage and recruit ATG16L1 to trigger xenophagy.	[49,120,123,124,125,130]
RIG-1 pathway	RIG-1 like receptors (RIRs), MAVS	Atg5-Atg12, Atg7, Beclin-1	Atg5, Atg12, and Atg7 negatively regulate RLR-dependent signaling. Atg5-Atg12 conjugates interact with RIG1 and MAVS and inhibit RLR-dependent antiviral signaling. RIG-1 RNA sensing pathway induces autophagy via a MAVS-TRAF6-Beclin-1 axis.	
CGAS-STING pathway	cGAS, cGAMP, STING, TRIM14	ULK1, Beclin-1, p62, LC3	cGAMP in STING pathway induces autophagy kinase ULK1, resulting in the inhibition of downstream signaling. cGAS interacts with Beclin-1 to enhance cytosolic microbial DNAs degradation via autophagy. TRIM14 stabilizes cGAS by recruiting USP14 and cleaving the lysine 48-linked ubiquitin, resulting in inhibition of p62-mediated autophagic degradation of cGAS. STING localized on the ER-Golgi body intermediate compartment (ERGIC) activates autophagy to eliminate DNA and viruses by inducing LC3 lipidation, which is dependent on ATG5 and WIPI2, not the ULK and VPS34 complex.	
